# Phosphocatalytic Kinome Activity Profiling of Apoptotic and Ferroptotic Agents in Multiple Myeloma Cells

**DOI:** 10.3390/ijms222312731

**Published:** 2021-11-25

**Authors:** Emilie Logie, Claudina Perez Novo, Amber Driesen, Pieter Van Vlierberghe, Wim Vanden Berghe

**Affiliations:** 1 Laboratory of Protein Science, Proteomics and Epigenetic Signaling (PPES) and Integrated Personalized and Precision Oncology Network (IPPON), Department of Biomedical Sciences, Campus Drie Eiken, University of Antwerp, Universiteitsplein 1, B-2610 Wilrijk, Belgium; emilie.logie@uantwerpen.be (E.L.); claudina.pereznovo@uantwerpen.be (C.P.N.); amber.driesen@uantwerpen.be (A.D.); 2 Department of Biomolecular Medicine, Ghent University, B-9000 Ghent, Belgium; pieter.vanvlierberghe@ugent.be

**Keywords:** ferroptosis, apoptosis, kinase, staurosporine, multiple myeloma, withaferin A

## Abstract

Through phosphorylation of their substrate proteins, protein kinases are crucial for transducing cellular signals and orchestrating biological processes, including cell death and survival. Recent studies have revealed that kinases are involved in ferroptosis, an iron-dependent mode of cell death associated with toxic lipid peroxidation. Given that ferroptosis is being explored as an alternative strategy to eliminate apoptosis-resistant tumor cells, further characterization of ferroptosis-dependent kinase changes might aid in identifying novel druggable targets for protein kinase inhibitors in the context of cancer treatment. To this end, we performed a phosphopeptidome based kinase activity profiling of glucocorticoid-resistant multiple myeloma cells treated with either the apoptosis inducer staurosporine (STS) or ferroptosis inducer RSL3 and compared their kinome activity signatures. Our data demonstrate that both cell death mechanisms inhibit the activity of kinases classified into the CMGC and AGC families, with STS showing a broader spectrum of serine/threonine kinase inhibition. In contrast, RSL3 targets a significant number of tyrosine kinases, including key players of the B-cell receptor signaling pathway. Remarkably, additional kinase profiling of the anti-cancer agent withaferin A revealed considerable overlap with ferroptosis and apoptosis kinome activity, explaining why withaferin A can induce mixed ferroptotic and apoptotic cell death features. Altogether, we show that apoptotic and ferroptotic cell death induce different kinase signaling changes and that kinome profiling might become a valid approach to identify cell death chemosensitization modalities of novel anti-cancer agents.

## 1. Introduction

Protein kinases (PKs) are enzymes that modify their substrate proteins by catalyzing the transfer of γ-phosphate from ATP to serine, threonine, and tyrosine residues. This process, known as protein phosphorylation, results in a functional change of the target protein and allows for a rapid signal transduction in response to intra- and extracellular triggers. Phosphorylation of proteins is considered to be one of the most crucial post-translational modifications and is required for proper regulation of various cellular functions, including metabolism [1,2], cell cycle regulation [3,4], differentiation [5,6], and cell death and survival [7,8,9]. Thus, it is not surprising that perturbed phosphorylation events or dysregulation of PKs often results in a diseased state, such as cancer. In multiple myeloma (MM), a hematological malignancy characterized by the uncontrolled proliferation of plasma cells, several kinases, including AKT and B-cell receptor signaling (BCR) kinases, are known to be involved in tumorigenesis and therapy resistance [10,11,12,13]. Targeting these malfunctioning kinases with protein kinase inhibitors (PKIs) could therefore offer significant clinical benefits to patients suffering from MM [14,15] or other cancer types [16]. Presently, 62 PKIs have been FDA-approved for clinical use and have had a major impact on the treatment of cancer worldwide [17]. However, no inhibitors have been approved for use in MM to date, mainly due to issues with drug efficacy [18]. Finding novel, targetable PKs in context of MM might aid in overcoming therapy resistance and curing the disease altogether.

Ferroptosis is a non-apoptotic mode of regulated cell death (RCD) that is orchestrated by an iron-dependent overproduction of lipid peroxides [19]. In the field of oncology, ferroptosis is currently being explored as a novel way to eradicate apoptosis-resistant cancer cells [20]. Different from apoptosis and other cell death modalities, ferroptosis does not depend on caspases or receptor-interacting protein 1, but heavily relies on the cysteine, lipid, and iron metabolism to promote cell death [21,22,23]. Taking into account that the majority of cancer types, including MM, are characterized by an increased iron uptake [24], an altered (cysteine) metabolism [25], and higher oxidative stress levels [26] compared to their healthy counterparts, ferroptosis-inducing agents might further destabilize these cellular processes to achieve complete tumor suppression [27]. However, the key signaling processes and determinants of ferroptotic cell death still largely remain elusive and need to be more extensively studied before the therapeutic use of ferroptotic kinase inhibitor compounds can fully be appreciated. Accumulating evidence indicates that PKs, such as AMPK and ATM, are involved in regulating ferroptosis [28,29]. Recent studies have even reported that PKI sorafenib is able to trigger ferroptosis in a variety of cell lines [30]. To this end, PKIs that inhibit a subset of kinases and subsequently induce ferroptotic cell death might hold promise as novel anti-cancer agents in therapy-resistant tumors, such as MM. Consequently, we performed phosphopeptidome kinase activity profiling of glucocorticoid (GC)-resistant multiple myeloma cells treated with either apoptotic or ferroptotic compounds to obtain cell death-specific inhibitory kinome activity signatures. This approach allows us to directly compare apoptosis- and ferroptosis-dependent kinase activity changes and to identify cell death type-specific kinases based on the differential peptide phosphorylation patterns. Finally, we also compared the kinome activity profile of natural anti-cancer agent withaferin A (WA) with the apoptotic and ferroptotic signatures to predict its cell death modality in MM cells.

## 2. Results

### 2.1. Both Apoptotic and Ferroptotic Cell Death Are Associated with a Progressive Inhibition of Protein Kinase Activity

**Figure 1:**
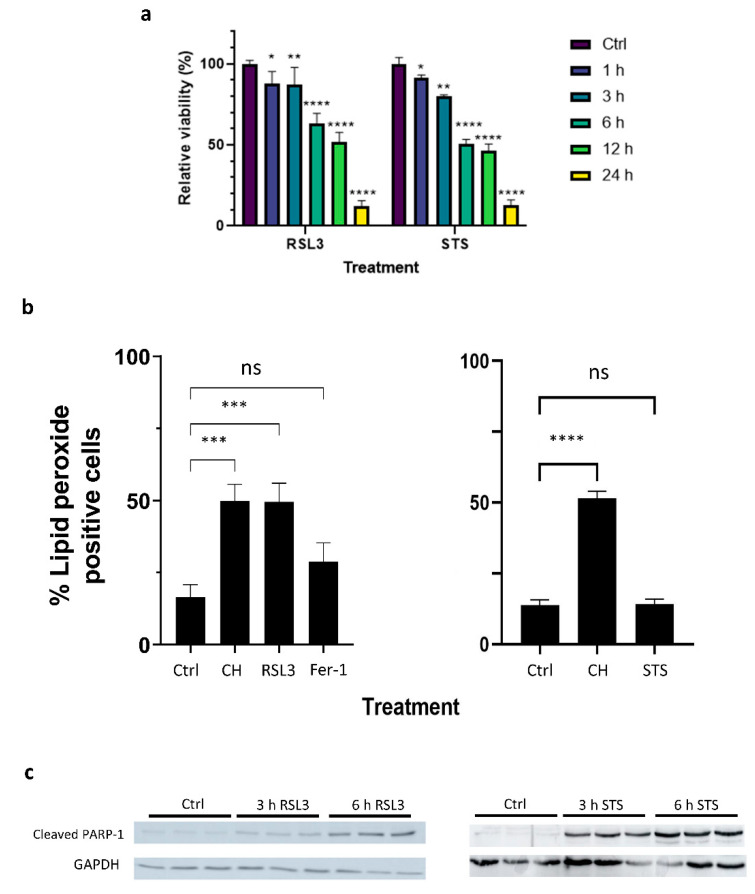
(**a**) Relative cell viability (%) of MM1R cells treated with 5 μM RSL3 (left), 1 μM STS (right), or untreated MM1R cells (Ctrl). (**b**) % MM1R cells treated with either 5 µM RSL3 (left) or 1 µM STS (right) undergoing a fluorescence shift from red (590 nm) to green (510 nm) as a consequence of increased lipid peroxidation. Cumene hydroperoxide (CH) is included as a positive control, while untreated controls (Ctrl) and cells pre-treated with ferroptosis inhibitor ferrostatin-1 and RSL3 (FRSL3) are included as a negative control. (**c**) Western blot detection and quantification of PARP-1 cleavage normalized against GAPDH expression levels in untreated MM1R cells (Ctrl) and MM1R cells treated with 5 µM RSL3 (left) or 1 μM STS (right) for 3 and 6 h. All data are presented as the mean ± s.d., n = 3 biologically independent samples per treatment (ns *p* > 0.05, * *p* < 0.05, ** *p* < 0.01, *** *p* < 0.001, **** *p* < 0.0001, ANOVA).

**Figure 2:**
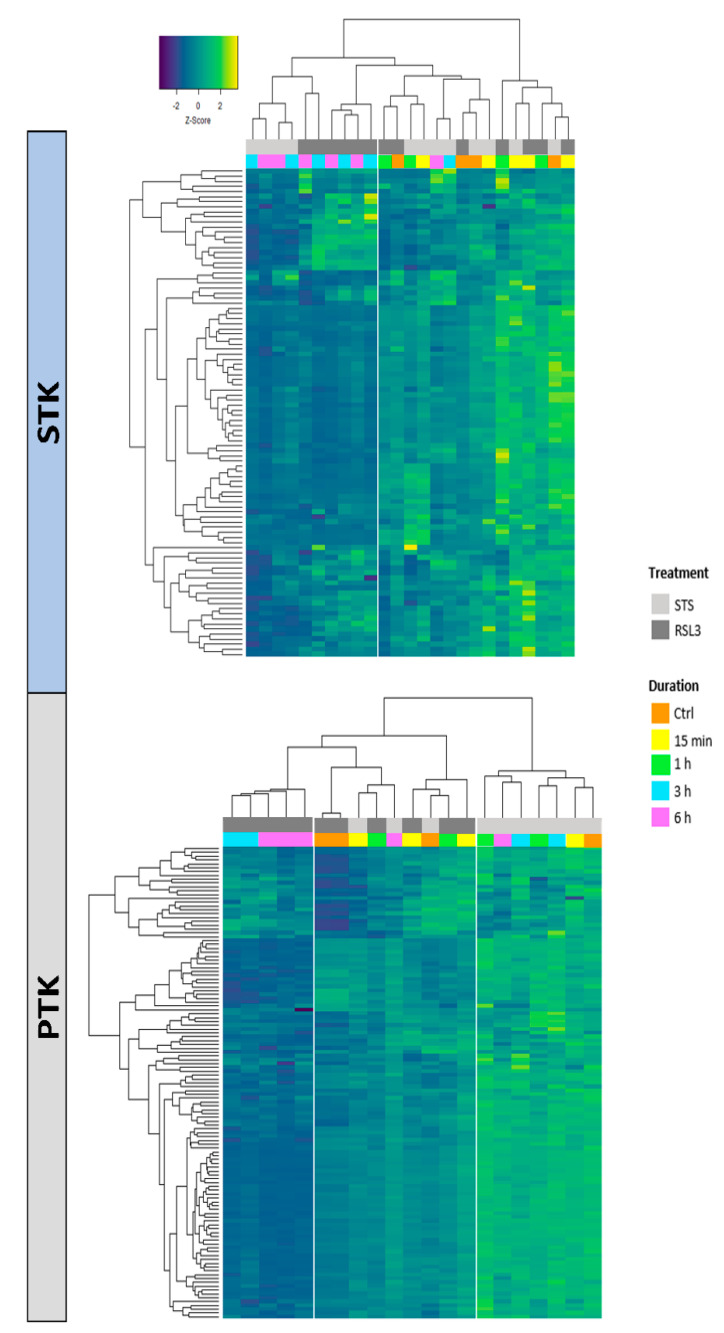
Heatmap visualization of individual phosphorylation intensities, represented as normalized z-scores, of peptides on serine/threonine kinase (STK) (upper panel) or tyrosine kinase (PTK) (lower panel) Pamchips which serve as substrates for serine/threonine or tyrosine kinases, respectively. Higher z-scores (yellow) indicate kinase activation while lower z-scores (dark blue) indicate kinase inhibition. MM1R cells were either left untreated (Ctrl) or treated with 5 μM RSL3 or 1 μM STS for increasing timepoints, as indicated by the figure legend.

Staurosporine (STS) is a reversible broad-spectrum protein kinase inhibitor that is widely used to induce apoptotic cell death in tumor models, including MM [31]. To characterize ferroptosis-dependent kinase signaling changes in GC-resistant multiple myeloma cells and compare them with apoptosis-dependent kinase changes, we analyzed the peptide phosphorylation profiles of STS-treated apoptotic and RSL3-treated ferroptotic MM1R cells. Both treatments steadily decreased cell viability of MM1R cells after ≥3 h treatment (Figure 1a) and were accompanied by either an increase in lipid peroxidation in ferroptotic cells or poly(ADP-ribose) polymerase 1 (PARP-1) cleavage in apoptotic cells (Figure 1b,c). In line with these observations, phosphorylation intensities of Ser/Thr kinase (STK) peptide substrates was markedly decreased after ≥3 h STS and RSL3 treatment (Figure 2). A similar trend could be observed for the tyrosine kinase (PTK) peptide substrates in RSL3-treated MM1R cells but not in STS-treated cells (Figure 2), suggesting that PTKs play a more pronounced role in ferroptotic cell death.

### 2.2. RSL3 and STS Treatment of MM1R Cells Mainly Affects CMGC and AGC Protein Kinases

**Figure 3:**
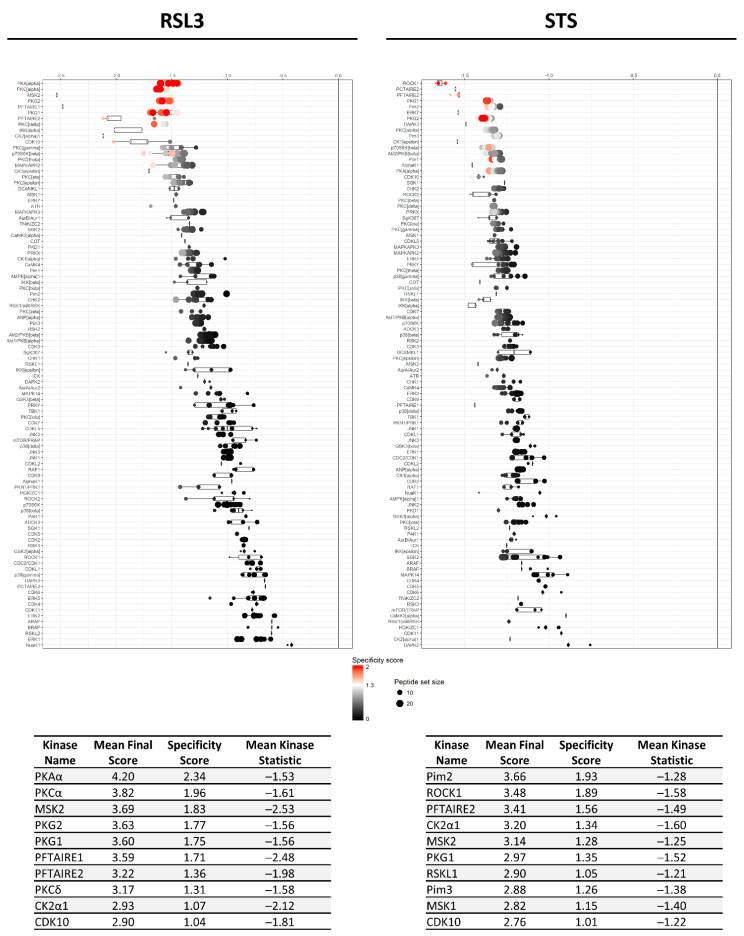
Mean final score plots of serine/threonine kinase (STK) activity profiling in MM1R cells treated with 5 μM RSL3 (**left**) or 1 μM STS (**right**) for 3 h. Plots display the predicted upstream STK ranked by their final score. The *x*-axis indicates the values for the normalized kinase statistic, which reflects the activity difference after treatment (e.g., negative values represent inhibited kinase activity). The size of each dot visualizes the size of the peptide set used for the upstream kinase prediction analysis. The color of the points depicts the specificity score, which indicates the specificity of the normalized kinase statistic with respect to the number of peptides used for predicting the corresponding kinase. Below each figure, a table displaying the top 10 ranked kinases and their kinase statistic and specificity score is presented. The final ranking of the kinases is based on the mean final score.

**Figure 4:**
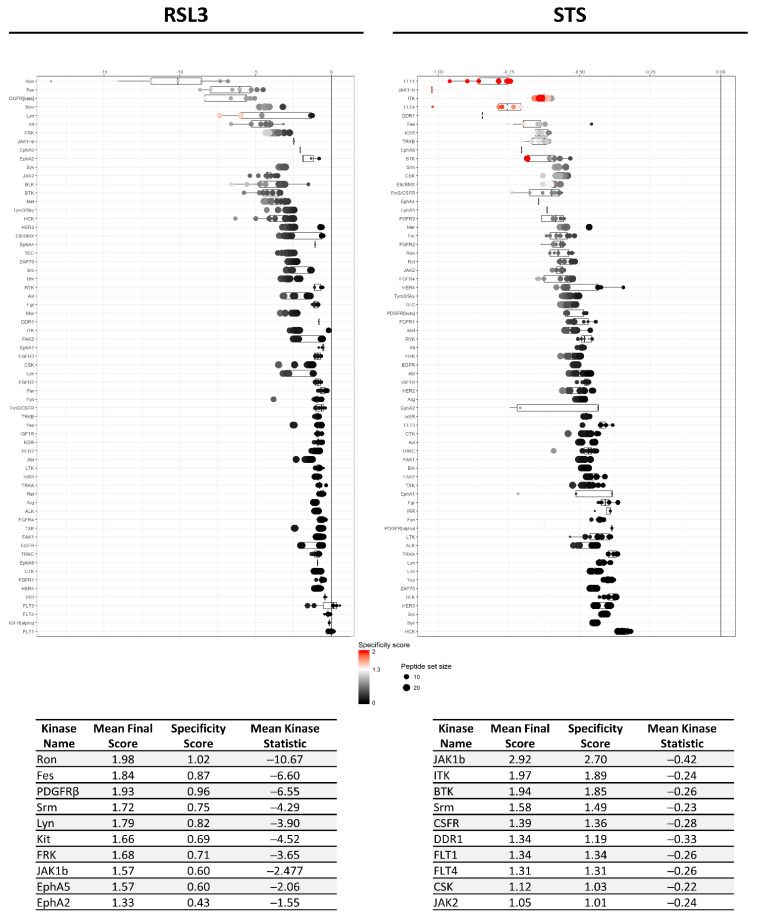
Mean final score plots of tyrosine kinase (PTK) activity profiling in MM1R cells treated with 5 μM RSL3 (**left**) or 1 μM STS (**right**) for 3 h. Plots display the predicted upstream STK ranked by their final score. The *x*-axis indicates the values for the normalized kinase statistic, which reflects the activity difference after treatment (e.g., negative values represent inhibited kinase activity). The size of each dot visualizes the size of the peptide set used for the upstream kinase prediction analysis. The color of the points depicts the specificity score, which indicates the specificity of the normalized kinase statistic with respect to the number of peptides used for predicting the corresponding kinase. Below each figure, a table displaying the top 10 ranked kinases and their kinase statistic and specificity score is presented. The final ranking of the kinases is based on the mean final score.

**Figure 5:**
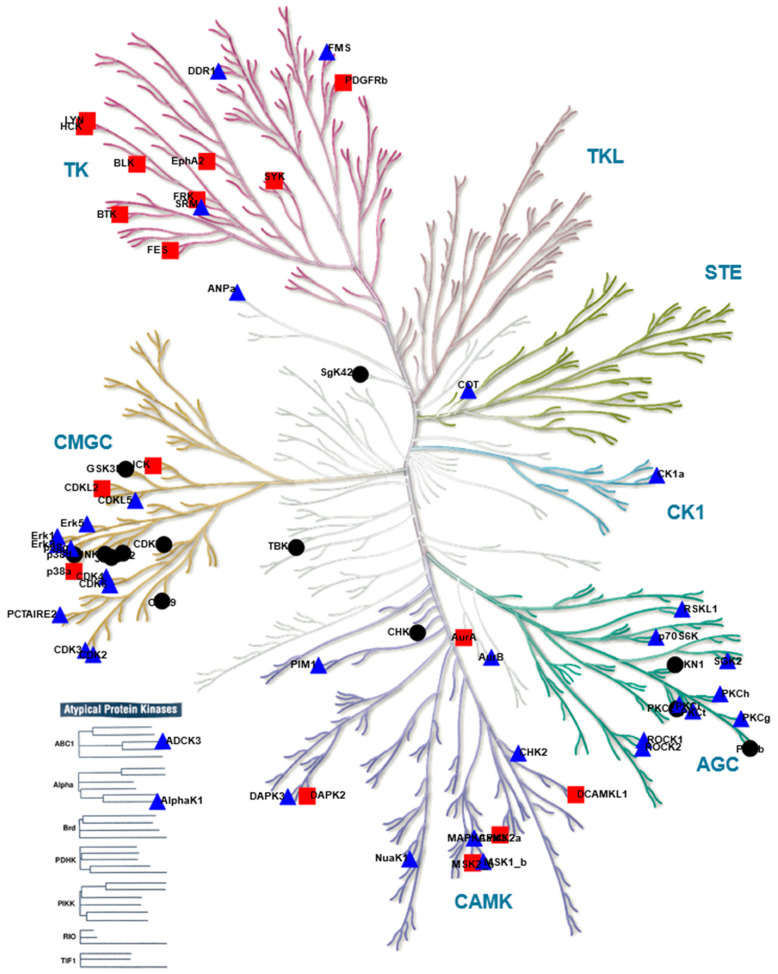
KinMap [38] phylogenetic kinome tree. Kinases inhibited by RSL3 treatment are indicated by red squares (■), kinases inhibited by STS treatment are marked as blue triangles (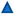), kinases inhibited by both compounds are indicated as black circles (•). Only kinases with a mean final score ≥ 1.3 are included in the graph. Abbreviations: TK, tyrosine kinase group; TKL, tyrosine kinase-like group; STE, serine/threonine kinase group; CK1, casein kinase 1 group; AGC, protein kinase A, G, and C group; CAMK, Ca^2+^/calmodulin-dependent kinase group; CMGC, cyclin-dependent kinase, mitogen-activated protein kinase, glycogen synthase kinase, and CDC-like kinase group. llustration reproduced courtesy of Cell Signaling Technology, Inc. (www.cellsignal.com, 27 September 2021).

Based on the differential peptide phosphorylation pattern that indicated that apoptosis- and ferroptosis-mediated cell death are associated with protein kinase inhibition (Figure 2, Supplementary Figure S1), upstream kinase (UPK) analysis was performed to identify the top 10 downregulated protein kinases under apoptotic and ferroptotic conditions after ≥3 h of treatment (i.e., the treatment time when a reduction in cell viability and inhibitory kinase effects are first observed as described in Section 2.1). The final STK and PTK ranking per condition is represented in Figure 3 and Figure 4, and is based on the mean final score of each kinase. Kinases with a mean final score of ≥1.3 and a kinase statistic of ≥0.5 were considered to be biologically relevant. Overall, kinase profiling revealed that both RSL3 and STS treatment of MM1R cells inhibits the activity of kinases involved in cell cycle regulation (PFTAIRE2, CK2α1, and CDK10), cytoskeletal reorganization (PKG1), cellular stress (MSK2), and inflammation (Srm, JAK1b) (Figure 2 and Figure 3). Most of these UPKs belong to the AGC or CMGC kinase group, implying that these kinase families in particular are important in regulating cell death in MM1R cells. This was further confirmed by phylogenetic kinase mapping which showed that the majority of STS- and RSL3-mediated downregulated kinases were enriched in the CMGC and AGC kinase families (Figure 5). Both compounds also target kinases classified into the CAMK kinase family, including aurora kinases (AurA, AurB) and death-associated protein kinases (DAPK) that are known to play a vital role in cell death regulation [32,33,34,35]. Remarkably, within the CAMK group, RSL3 and STS can target different members within the same family. For instance, while RSL3 can to significantly inhibit DAPK2 and AurA activity, STS preferentially inhibits DAPK3 and AurB. Further exploration of the synergistic interaction between apoptosis and ferroptosis inducers in multiple myeloma treatment might therefore help to identify effective combination therapies. A prior study in pancreatic tumor cells has revealed, for example, that ferroptotic agents can sensitize cells to TRAIL-induced apoptosis [36].

**Figure 6:**
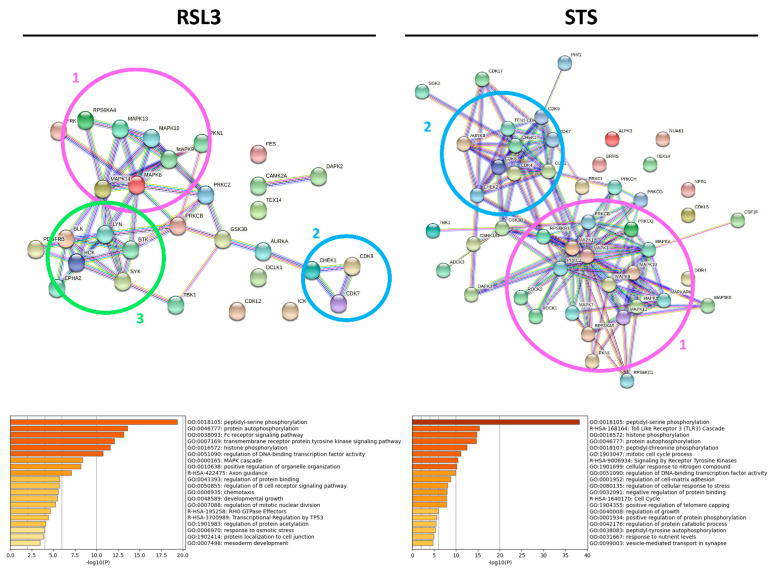
Network interaction and functional enrichment analysis of predicted kinases inhibited by RSL3 (**left**) or STS (**right**) in MM1R cells. The top panel visualizes the protein interaction networks of kinases with a mean final score ≥1.3, generated with String (v11) [39]. The clusters visible in the network are marked by a circle and include a MAPK signaling cluster (pink circle, number (1), CDK signaling (blue circle, number (2), and B-cell receptor signaling (green circle, number (3). The lower panel shows the Metascape [40] functional enrichment analysis of the included protein kinases.

Although STS and RSL3 display similarities in their inhibitory kinase profiles in MM1R cells, key differences between both treatment conditions exist as well. Compared to RSL3, STS can to inhibit a broader range of STKs, including kinases belonging to the CK1 (CK1α), STE (COT), and Atypical (AlphaK1, ADCK3) families. Even within the AGC and CMG families, the number of kinases targeted by STS exceeds the number of RSL3-inhibited kinases (Figure 5). In contrast, RSL3 treatment has a more pronounced effect on PTKs and is able to inhibit several kinases that are involved in BCR signaling, which we previously reported to be hyperactivated in therapy-resistant MM cells [10]. Network interaction and functional enrichment analysis indeed demonstrates that kinase inhibitory changes mediated by RSL3, but not by STS, are enriched in regulation of BCR signaling activity (Figure 6). This might explain why some B-cell malignancies, including diffuse large B-cell lymphoma, are more prone to ferroptosis-induced cell death in comparison to other solid tumors [37].

### 2.3. Predicting the Cell Death Modality of Withaferin a Based on Kinome Profiling

After characterizing the kinase changes taking place in apoptotic STS-treated and ferroptotic RSL3-treated MM1R cells, we wondered whether the kinase inhibitory profile of these compounds can be used to predict the cell death modality of other anti-cancer agents. Our findings from a previous study demonstrate that the natural steroidal lactone WA effectively kills GC-resistant MM1R cells and that the induced cell death is, in part, orchestrated by WA-mediated inhibition of Bruton tyrosine kinase (BTK), a key player in BCR signaling [10]. However, whether this WA-mediated BTK-inhibition promotes apoptosis, ferroptosis, or other cell death modalities in MM1R cells has not been elucidated yet. To this end, we treated MM1R cells with WA and compared its kinase profile with STS and RSL3. Similar to STS and RSL3, a time-dependent decrease in cell viability could be observed in MM1R cells treated with 1 μM WA (Supplementary Figure S2) and was associated with a decrease in peptide phosphorylation after ≥3 h of treatment (Supplementary Figure S3).

**Figure 7:**
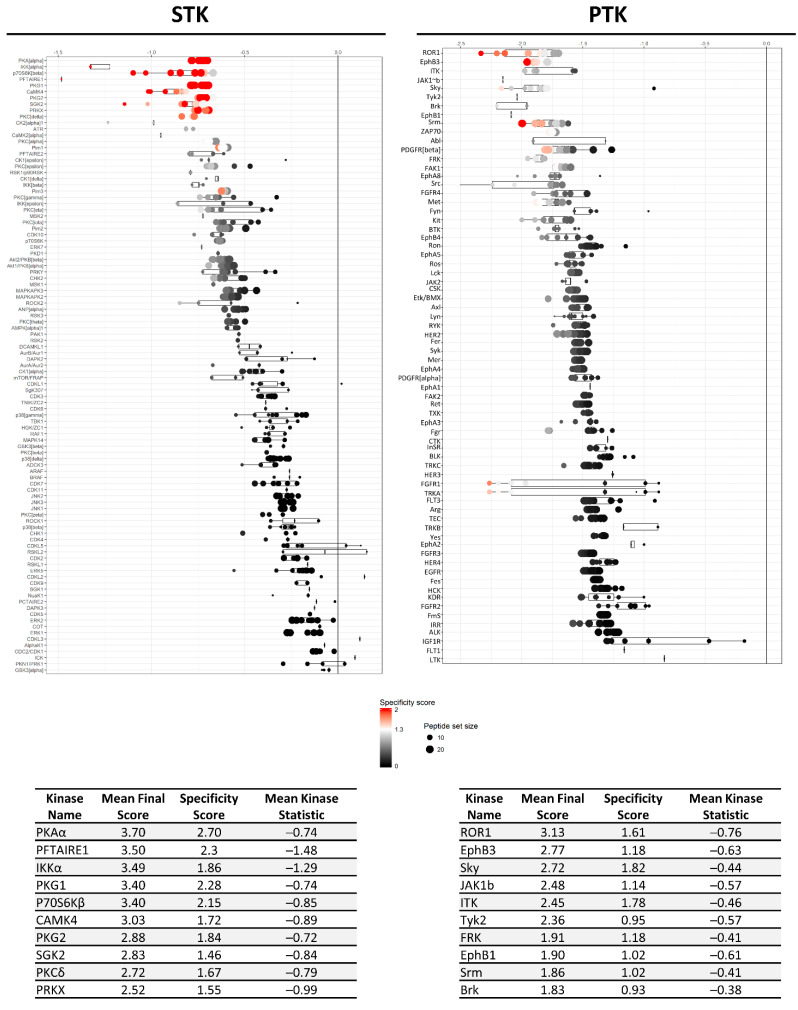
Mean final score plots of serine/threonine (STK) and tyrosine kinase (PTK) activity profiling in MM1R cells treated with 1 μM withaferin a (WA) 3 h. Plots display the predicted upstream STK ranked by their final score. The *x*-axis indicates the values for the normalized kinase statistic, which reflects the activity difference after treatment (e.g., negative values represent inhibited kinase activity). The size of each dot visualizes the size of the peptide set used for the upstream kinase prediction analysis. The color of the points depicts the specificity score, which indicates the specificity of the normalized kinase statistic with respect to the number of peptides used for predicting the corresponding kinase. Below each figure, a table displaying the top 10 ranked kinases and their kinase statistic and specificity score is presented. The final ranking of the kinases is based on the mean final score.

**Figure 8:**
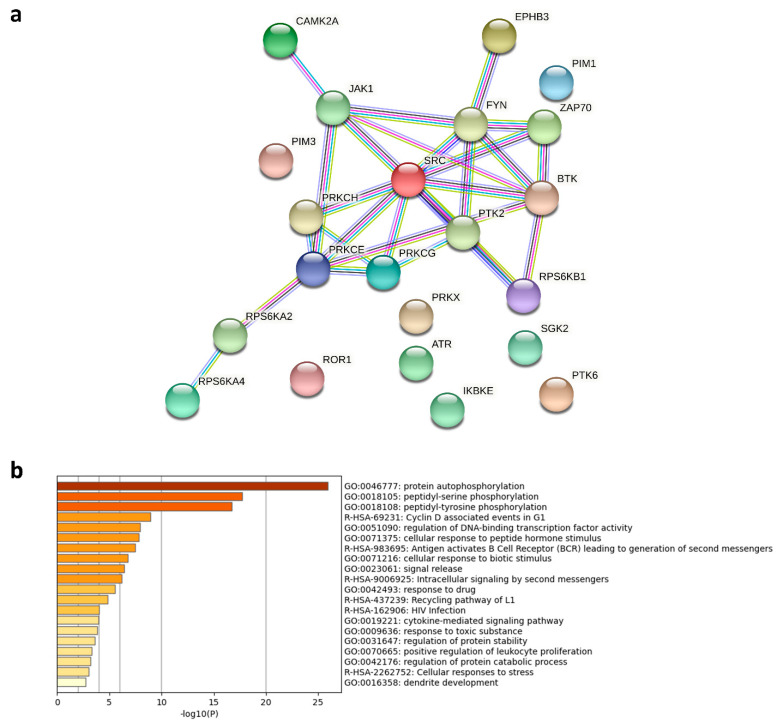
(**a**) Network interaction analysis of predicted kinases inhibited by 1 μM Withaferin a (WA) in MM1R cells. The protein interaction network (generated with String (v11) [39]) includes all kinases inhibited by WA with a mean final score ≥1.3. (**b**) Metascape [40] functional enrichment analysis of the protein kinases included in the protein interaction network.

Further UPK analysis revealed that the top 10 WA-inhibited STKs and PTKs include PKα, PFTAIRE1, PKG2, PKCδ, and FRK (Figure 7), which are mainly targeted by RSL3, but not STS. Likewise, network interaction and functional enrichment analysis demonstrated that WA resembles RSL3 in its ability to inhibit B-cell receptor signaling by inhibiting BTK and other PTKs (Figure 8a,b), which is in line with our previous observations [10]. On the other hand, phylogenetic kinome mapping showed that WA is also able to target a broad range of STKs, especially those classified into the AGC kinase family, suggesting that the inhibitory kinome profile of WA is reminiscent of the STS profile as well (Supplementary Figure S6). PKCη, PKCε, PKCγ, p70S6K, and SGK2 are all AGC kinases that are inhibited by both WA and STS. Based on the duality of its kinome profile, WA seems to be able to both induce apoptotic and ferroptotic cell death. In agreement with this observation, previous studies have reported that WA is able to trigger different cell death modalities based on the cellular context and concentration range used [41,42].

Despite its similarities with RSL3, we observed no increase in lipid peroxidation in MM1R cells after treatment with WA (data not shown). WA-induced cell death could also not be reversed by pre-treatment of MM1R cells with ferrostatin-1, an inhibitor of ferroptosis (Supplementary Figure S4). In contrast, apoptosis inhibitor Z-VAD-FMK was able to partially rescue WA-mediated cell death, suggesting that WA triggers apoptosis rather than ferroptosis in MM1R cells (Supplementary Figure S4). Indeed, and increase in PARP-1 could also be observed in MM1R cells upon prolonged WA exposure (Supplementary Figure S5). Taken together, our results indicate that the inhibitory kinome profile of WA is similar to apoptotic and ferroptotic kinome profiles, suggesting that WA is able to promote both of these cell death mechanisms. Although WA and RSL3 are both able to inhibit BTK and BCR signaling, WA seems to trigger apoptosis rather than ferroptosis in MM1R cells. Most likely, other WA-targeted pathways, including NF-κB signaling [43], proteasome-mediated protein degradation [44], and heat shock-regulated stress signaling [45], tip the cell death balance towards apoptosis. It therefore remains important that kinase activity changes are interpreted within a broader biological kinome context.

## 3. Discussion

It is estimated that one quarter of all drug discovery efforts aim to target PKs, making them the largest group of clinical drug targets in the field of oncology [17]. Although several PKIs have reached phase II of clinical trials for treatment of MM, none have reached the market yet [46]. This is somewhat remarkable given that both myelomagenesis and development of therapy resistance are, at least in part, associated with perturbations in protein phosphorylation [47]. Both hyperactivation of the PI3K/AKT/mTOR and JAK/STAT signaling pathways, for example, are associated with poor prognosis and MM tumor proliferation, and are actively being explored in preclinical drug research as potential drug targets [48,49]. However, since neither AKT or STAT inhibitors have been FDA-approved for clinical use in MM, identifying other targetable PKs crucial in MM tumorigenesis might contribute to the development of novel anti-MM compounds. Because accumulating evidence suggest that B-cell malignancies, including diffuse large B-cell lymphoma (DLBCL) and MM, are sensitive to ferroptotic cell death [37], finding kinases essential for ferroptotic cell death may offer translational potential for MM treatment by FDA approved kinase inhibitors. To this end, we applied phosphopeptidome kinome activity profiling of RSL3-treated GC-resistant MM1R cells to identify kinases significantly inhibited by ferroptosis. We also compared this ferroptosis kinase signatures with those of STS-treated apoptotic MM1R cells to determine to which end each cell death modality has its own kinome profile. Our analysis revealed that prolonged treatment (≥3 h) with either STS or RSL3 resulted in an overall decrease in STK activity, which was associated with a time-dependent increase in cell death. A similar inhibitory effect on PTKs was observed only in MM1R cells exposed to RSL3 exposure, implying that PTKs are more crucial in ferroptosis signaling compared to apoptosis signaling. This was also observed in our UPK analysis of the differential peptide phosphorylation patterns, where the mean kinase statistic score of top predicted RSL3 inhibited kinases was much higher compared to the score of STS inhibited kinases. Remarkably, functional enrichment analysis indicated that the majority of RSL3-targeted PTK kinases are involved in BCR, crucial for proliferation and survival of B-cells. This could possibly explain why B-cell cancers are more prone to ferroptotic cell death, as they heavily rely on BCR-related kinases for mediation of therapy resistance and tumor proliferation [13,50,51]. Although inhibition of BCR signaling is typically associated with apoptosis induction in several B-cell malignancies [52,53,54], this only has been sporadically explored in MM and deserves further investigation [55]. Alternatively, most BCR-related kinases are known to play a variety of different functions depending on their cellular context. Syk, for example, also orchestrates TNFα and NF-κB signaling in MM and other, non-hematological cells [55,56,57]. By inhibiting Syk, GPX4-inhibitor RSL3 potentially triggers MM cell death through inactivation of these two oncogenic pathways. In line with this hypothesis, a recent genetic-based kinome screening against ferroptosis in MDA-MB-231 breast cancer cells, revealed an important role for Syk and other TNFα/NF-κB kinase players suggesting that Syk-mediated inhibition by ferroptotic-inducing agents might be a universal phenomenon [29]. Indeed, a study in mucosal samples from ulcerative colitis patients revealed that NF-κB inhibition promotes ferroptotic cell death, albeit that the involvement of Syk was not investigated in this experimental setup [58]. Finally, another plausible explanation is that especially inhibition of PDGFRβ, in this study predicted to be the third most significant inhibited PTK by RSL3, is important to induce ferroptosis in MM. PDGFRβ is one of the major targets of sorafenib [59], a clinically approved PKI for treatment of hepatocellular and advanced renal cancer [60] that has been reported to induce ferroptosis in a variety of different human cell lines [30]. Although the correlation between ferroptosis and sorafenib has primarily been linked to non-kinase related events [61,62], the role of PDGFRβ in RSL3-dependent cell death has not been explored. Intriguingly, overexpression of PDGFRβ is typically associated with worse prognosis in leukemia and myeloma patients [63,64].

Compared to RSL3, the inhibitory effect of STS on STKs is considerably larger. As revealed by phylogenetic mapping STS targets STKs from different kinase families, including the STE, CK1, Atypical, CAMK, AGC, and CMGC families. This is in line with previous studies that have identified an extensive range of STS-inhibited kinases in HepG2 liver cells, such as PGK1, AurB, PKAs, PKCs, and CDKs [65,66,67]. However, a subset of these kinases are also significantly inhibited by RSL3, highlighting that different cell death modalities can have overlapping kinome profiles. Most of the common kinases belong to the CMGC and AGC kinase families and include PFTAIRE2, CK2α1, CDK10, PKG1, and MSK2. Taking into account that the CMGC and AGC kinases mainly regulate cell cycle progression, proliferation, and survival, apoptosis- and ferroptosis mediated inhibition of these kinases seems crucial in promoting cell death. Both STS and RSL3 also impact CAMK kinases, which are involved in crucial cellular functions as well. Some CAMKs, such as CAMKK2 and CAMK4, have recently been associated with ferroptotic cell death [68,69]. Although these kinases were not significantly inhibited by RSL3 in our setup, we did reveal that CAMK2A activity was considerably lower in ferroptotic MM1R cells, hinting that a larger CAMK-signaling network may be involved in ferroptosis signaling. Again, these observations are in line with the aforementioned genetic-based kinase screen, which also revealed CAMK2A to be one of the most crucial ferroptosis-dependent kinases [29]. However, results from the genetic-based screen also identified ATM and ATR as key ferroptosis regulators. Our phosphopeptidome screening did also point towards a RSL3-mediated inhibition of ATR, although this inhibition was found not to be biologically relevant as the threshold of mean kinase score ≥1.3 was not reached (0.55). Similarly, we did not find a strong association between RSL3-induced ferroptosis and AMPK activity (0.24) although this kinase has previously been associated with ferroptotic cell death [28]. These discrepancies can partly be explained by differences in experimental setup, as various cell lines, ferroptosis inducers, and treatment times have been used throughout the listed studies. As kinase expression and kinase-phosphatase interplay is highly dependent on cellular context, repeating the current study in other (MM) cell lines or tissues with similar experimental conditions is therefore vital to further determine which kinase are truly essential for RSL3-mediated ferroptosis. Furthermore, the predicted kinome activity profiles identified here should be further experimentally validated by means of western blotting, phosphoproteomics, NanoBRET kinase assays, and silencing approaches to further support their biological relevance.

To preliminary determine whether the obtained ferroptosis and apoptosis kinome activity profiles can be applied to predict therapeutic efficacy and/or characterize cell death properties of novel anti-cancer agents, we also treated MM1R cells with WA, a compound reported to inhibit several PKs (reviewed in [70]). Remarkably, WA can to trigger different pro-apoptotic and pro-ferroptotic signaling pathways in a dose- and cell context-dependent manner [41,71,72,73,74]. We found that these promiscuous anti-cancer properties of WA were also mirrored in its differential phosphopeptidome and kinome activity profile, which portrayed similarities to both the apoptosis and ferroptosis signatures. Functional enrichment and UPK analysis showed that the top WA-inhibited kinases mostly overlapped with RSL3-targeted kinases and that these were also enriched in regulation of BCR signaling. However, phylogenetic tree visualization also demonstrated that, like STS, WA can to target a broad range of ACG kinases, including PKCη, PKCε, PKCγ, p70S6K, and SGK2. These results are in agreement with previous studies reporting WA-mediated inhibition of BCR-related kinases [10], PKC kinases [75], and p70S6K [76]. WA also targets a subset of kinases, including IKK, that are not affected by STS nor by RSL3 treatment, revealing that WA also has unique inhibitor effects on kinase signaling in MM. This might explain why, despite its similarities with RSL3, WA triggers apoptosis and not ferroptosis in MM1R cells, as indicated by cell viability, western blot, and lipid peroxidation analysis. For example, inhibition of IKK by WA in DLBCL is described to promote apoptosis [77] and might favor the WA-triggered cell death modality in MM1R cells towards apoptotic cell death. Likewise, WA-mediated targeting of non-kinase signaling pathways, such as proteasome degradation and unfolded protein stress responses [78,79], might further impact cell death faith. Alternatively, the occurrence of lipid peroxidation might be crucial for some of the ferroptosis-driven promiscuous inhibitory effects observed in MM1R cells. Lipid peroxidation directly impacts the cellular redox status and can, in turn, inhibit tyrosine kinases by (reversible) cysteine oxidation [80,81,82]. Thus, although both WA and RSL3 possess chemical moieties that can interact with proteins harboring nucleophilic active sites, including (seleno)cysteine sites [10,37], lipid peroxidation does not take place in WA-treated cells and might be the main explanation why apoptosis, rather than ferroptosis, is the main WA cell death modality in MM cells.

The results presented in this study demonstrate that STS, RSL3, and WA inhibit kinase activity of multiple kinases and lack target specificity. Although this can drastically impact the clinical applicability of these polypharmacological compounds as kinase inhibitors, their multi-target pharmacological profile might offer some advantages as well. In contrast to the target specificity paradigm, a dominant concept in drug discovery, multi-targeting protein kinase inhibitors could simultaneously affect different nodes within a kinase signaling network and more effectively eliminate therapy-resistance tumor cells [83]. Combination treatments of PI3K and EGFR inhibitors have, for example, shown to be effective in different disease models and are currently being tested in clinical settings [84,85,86]. Similarly, dual inhibition of PI3K and mTOR by one or more PKIs have demonstrated to be more effective in glioma treatment than inhibition of either target alone [87]. These data imply that broad-spectrum kinase inhibitors hold promise in certain oncological settings, and might improve response rates in therapy resistant tumors, such as MM. However, beneficial drug effects need to be carefully balanced again adverse, toxic effects and this requires extensive pharmacological profiling of drug candidates. In case of WA, previous work has shown that both covalent and non-covalent interactions are responsible for the promiscuous WA-mediated inhibition of (BCR-related) kinases [10]. Further molecular structure optimization or functional silencing approaches with shRNA libraries could aid in distinguishing druggable key anti-MM targets from adverse off-targets [88]. The molecular mechanism by which RSL3 affects kinase activity is far less characterized. Although several kinases, including ATM and AMPK, have been linked to ferroptotic cell death [28,29,68], it remains largely unknown how RSL3 mediates inhibition of protein kinases. Possibly, RSL3 indirectly influences kinase activity by increasing cellular oxidative stress and lipid peroxidation although this deserves further investigation [89,90,91,92].

In conclusion, ferroptosis and apoptosis inducers both elicit a broad-spectrum PK inhibitor signaling profile in MM1R cells. Although both cell death modalities display similarities in their inhibitory kinome activity profile, unique signatures in PK targeting could also be detected. These unique peptide-based kinase activity profiles might aid in identifying novel (ferroptosis-based) kinase targets in MM treatment or might help stratification of other apoptosis or ferroptosis sensitizing anti-cancer compounds. Moreover, multi-targeting of apoptosis- and ferroptosis-specific kinases might prove to be more efficient in treating multifactorial (therapy-resistant) diseases, such as cancer. Future studies should aim to further validate ferroptosis-specific kinases in different experimental models of MM to ultimately pinpoint which FDA approved PKIs potentially offer the best clinical benefits.

## 4. Materials and Methods

### 4.1. Cell Culture and Cell Viability Assays

Human MM1R cells (CRL-2975) were purchased from ATCC (Manassas, VA, USA) and grown in RPMI-1640 medium supplemented with 10% FBS (E.U Approved; South American Origin) and 1% Pen-Strep solution (Invitrogen, Carlsbad, CA, USA) at 37 °C in 5% CO_2_. Cell viability was measured with the colorimetric 3-(4,5-dimethylthiozol-2-yl)-2,5-diphenyltetrazolium bromide (MTT) assay (Sigma Aldrich, St. Louis, MO, USA) as previously described [93]. In all experiments, untreated MM1R cells were used as control cells.

### 4.2. Antibodies and Reagents

STS and RSL3 were purchased from Selleckchem (Houston, TX, USA), dissolved in DMSO and stored as 50 mM stocks at −20 °C. Antibodies targeting cleaved PARP-1 (sc-7150) and GAPDH (2118S) were obtained from Santa Cruz Biotechnology (Dallas, TX, USA) and Cell Signaling Technology (Danvers, MA, USA), respectively.

### 4.3. Lipid Peroxidation Assay

Cellular lipid reactive oxygen species were measured using the Image-iT™ Lipid Peroxidation Kit (C10445, ThermoFisher Scientific, Waltham, MA, USA) according to the manufacturer’s protocol. In short, cells were seeded in 6 well plates at a density of 5 × 10^5^ cells/well and treated the next day with 5 μM RSL3 (with or without pre-treatment with 2 μM Fer-1) or 100 μM cumene hydroperoxide (positive control). Cells were subsequently incubated for 30 min with 10 μM Image-iT™ Lipid Peroxidation Sensor at 37 °C. After incubation, cells were collected by trypsinization with TrypLE Express Enzyme (ThermoFisher Scientific, Waltham, MA, USA). Cells were washed 3 times with pre-warmed PBS and fluorescence shift from 590 nm to 510 nm was measured with the CytoFlex flow cytometer (Beckman Coulter Life Sciences, Indianapolis, IN, USA). Finally, the 510/590 ratio was calculated and visualized as a % of lipid peroxide positive cells.

### 4.4. Protein Extraction and Western Blot Analysis

Cellular protein extraction occurred by resuspending cell pellets in 0.5 mL RIPA buffer (150 mM NaCl, 0.1% Triton X-100, 1% SDS, 50 mM Tris-HCl pH 8) supplemented with PhosphataseArrest (G-Biosciences, Saint-Louis, MO, USA) and protease inhibitors (Complete Mini^®^, Roche), Basel, Switzerland). After 15 min incubation on ice with regular vortexing, samples were briefly sonicated (1 min, amplitude 30 kHz, pulse 1 s) and centrifuged at 13,200 rpm for 20 min at 4 °C. Solubilized proteins were transferred to new Eppendorf tubes and stored at −20 °C. Protein lysates were separated using Bis-Tris SDS-PAGE with a high-*M*_W_ MOPS running buffer, and transferred onto nitrocellulose membranes (Hybond C, Amersham) using the Power Blotter System (Thermofisher, Waltham, MA, USA). Blocking the membranes for 1 h with blocking buffer (20 mM Tris-HCl, 140 mM NaCl, 5% BSA, pH 7.5) at RT was followed by overnight incubation with the primary antibody at 4 °C. Blots were then incubated for 1 h with the secondary, HRP dye-conjugated antibody (Dako, Glostrup, Denmark) after which chemiluminescent signals were detected with the Amersham Imager 680 (Cytiva, Marlborough, MA, USA) and quantified with the ImageJ software (v1.53j, National Institutes of Health, Bethesda, MD, USA) [94].

### 4.5. Kinase Activity Profiling Using PamChip^®^ Peptide Microarrays

Kinase activity profiling was performed as previously described [10]. In short, MM1R cells were treated with 1 μM STS or 5 μM RSL3 for increasing time periods (*n* = 3 biologically independent samples per time point) and lysed in M-PER lysis buffer (Mammalian Extraction Buffer) containing 1:100 Halt protease and phosphatase inhibitor cocktail (78440, Thermofisher, Waltham, MA, USA). Protein yield was determined using the Pierce™ BCA Protein Assay method (23225, Thermo Scientific) [95]. Tyrosine and serine-/threonine kinase profiles were determined by employing the PamChip^®^ peptide microarray system (Pamgene, s-Hertogenbosch, The Netherlands) according to the manufacturer’s instructions. UPK analysis was automatically performed by the BioNavigator Analysis software tool (Pamgene, s-Hertogenbosch, The Netherlands) based on the tyrosine and serine/threonine kinase phosphorylation patterns. Each predicted kinases is scored with a mean kinase statistic, a specificity score and a mean final score. The mean kinase statistic score depicts the effect size (values) and direction (+ or −) between the group difference, while the specificity score indicates the specificity of the normalized kinase statistics with respect to the amount of peptides used for predicting the corresponding kinase. The mean final score is typically used to generate the ranked predictive list of kinases and is a combination of the specificity score and the group difference.

Kinase interaction networks and phylogenetic trees were generated using the String database (v11) [39] and the KinMap web-based tool [38], respectively. These visualizations were performed with all kinases with a mean final score of ≥1.3, as recommended by Pamgene. Functional enrichment analysis was performed with Metascape using the default settings [40].

### 4.6. Statistical Analysis

Statistical tests were performed in GraphPad Prism (v7.0) (GraphPad Software, San Diego, CA, USA) unless otherwise stated in the main text. Results were considered to be statistically significant when *p*-values < 0.05 were obtained.

## Data Availability

The data presented in this study are available in this article.

## Supplementary Materials

The following are available online at https://www.mdpi.com/article/10.3390/ijms222312731/s1.
