# Supplementary material for: Phosphocatalytic Kinome Activity Profiling of Apoptotic and Ferroptotic Agents in Multiple Myeloma Cells
Source: Int J Mol Sci. 2021 Nov 25;22(23):12731. doi: 10.3390/ijms222312731 (PMC8657914; doi:10.3390/ijms222312731)
Supplement: Supplementary file 1 [file ijms-22-12731-s001.zip › ijms-1417519-supplementary.pdf]

Supplementary Materials

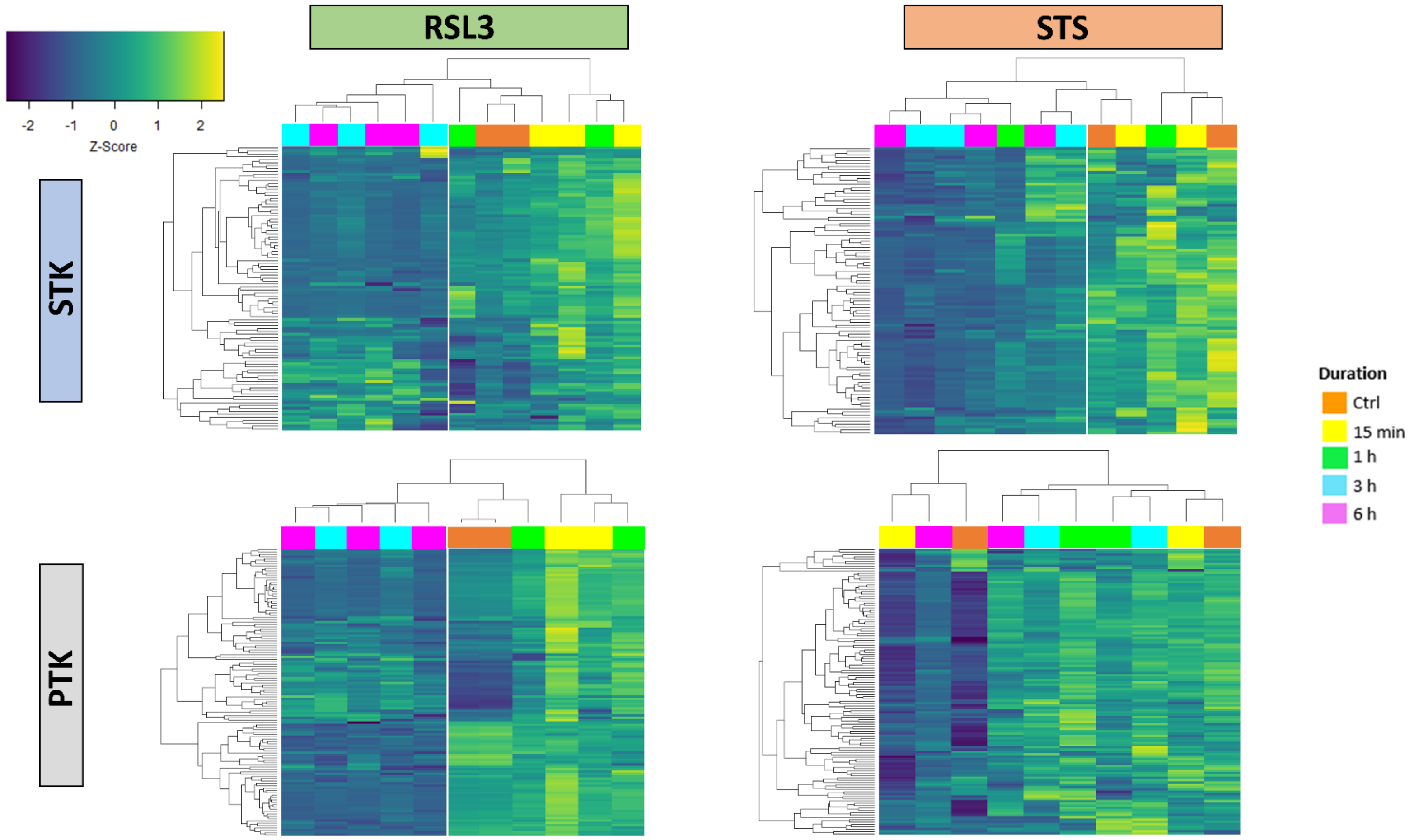

Figure S1. Heatmap visualization of individual phosphorylation intensities, represented as normalized z-scores, of peptides on serine/threonine kinase (STK) (upper panel) or tyrosine kinase (PTK) (lower panel) chips which serve as substrates for serine/threonine or tyrosine kinases, respectively. Higher z-scores (yellow) indicate kinase activation while lower z-scores (dark blue) indicate kinase inhibition. MM1R cells were treated with either 5 µM RSL3 (left panel) or 1 µM STS (right panel) for increasing timepoints, as indicated by the figure legend.

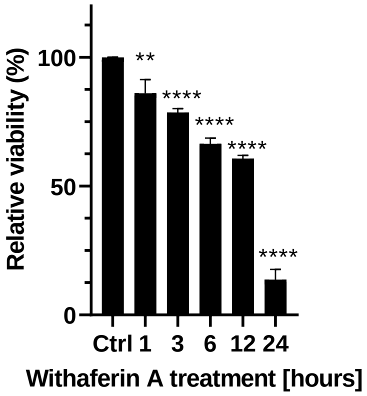

Figure S2. Relative cell viability (%) of MM1R cells treated with 1 μM WA. Data are presented as the mean ± s.d., n=3 biologically independent samples per treatment (**p < 0.01, ****p < 0.0001, ANOVA).

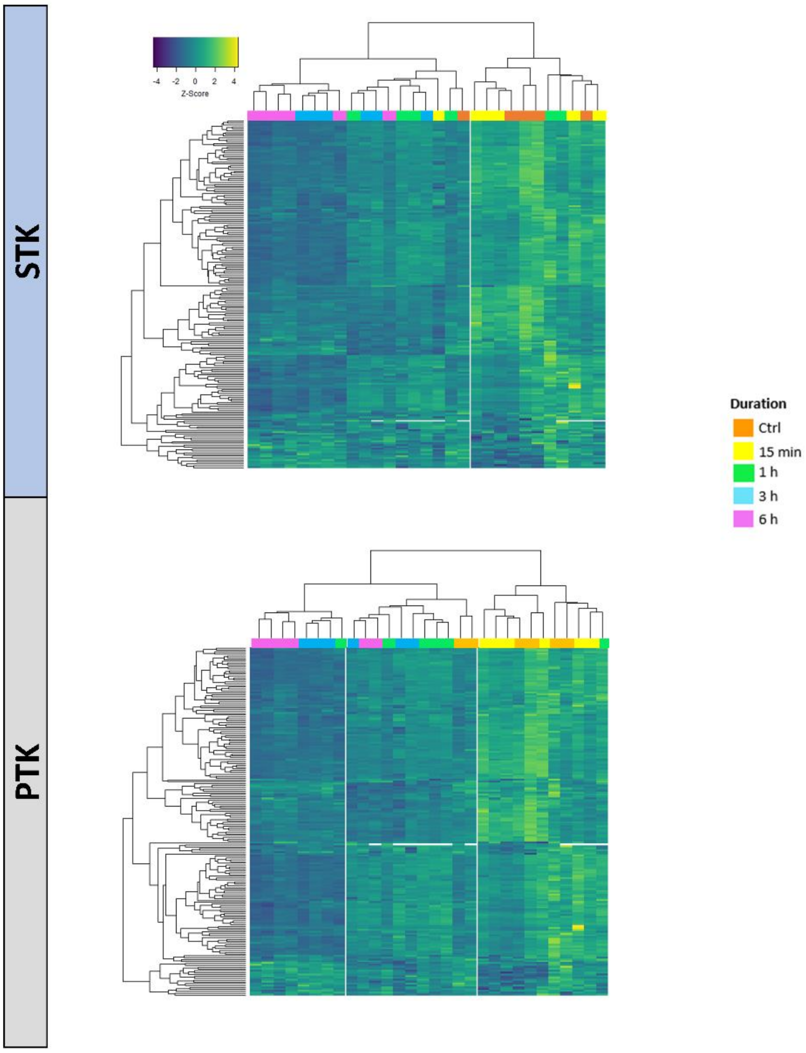

Figure S3. Heatmap visualization of individual phosphorylation intensities, represented as normalized z-scores, of peptides on serine/threonine kinase (STK) (upper panel) or tyrosine kinase (PTK) (lower panel) chips which serve as substrates for serine/threonine or tyrosine kinases, respectively. Higher z-scores (yellow) indicate kinase activation while lower z-scores (dark blue) indicate kinase inhibition. MM1R cells were treated with 1 μM Withaferin A for increasing timepoints, as indicated by the figure legend.

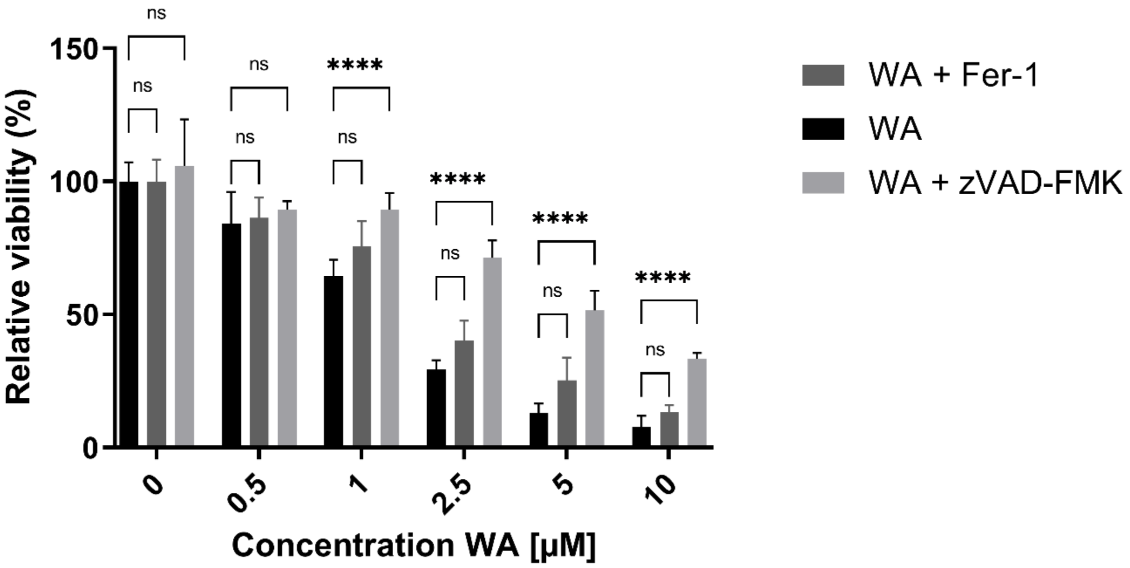

Figure S4. Relative cell viability (%) of MM1R cells treated with 1 µM WA for 24 hours with or without 2 hour pre-treatment with 2 µM ferrostatin-1 (Fer-1) or 50 µM zVAD-FMK. Data are presented as the mean ± s.d., n=3 biologically independent samples per treatment (ns p > 0.05, ****p < 0.0001, ANOVA).

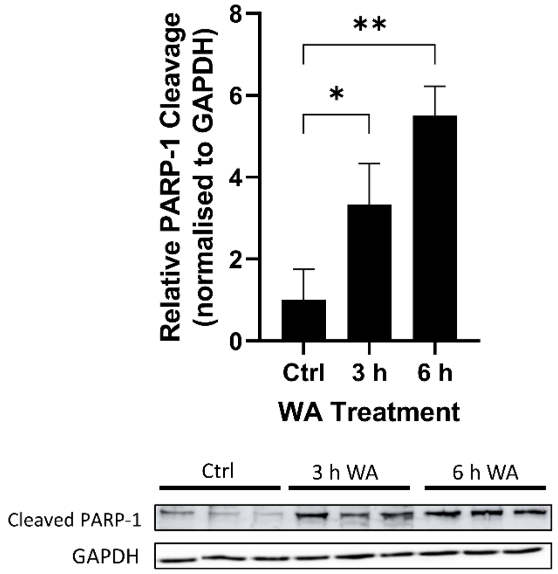

Figure S5. Western blot detection and quantification of PARP-1 cleavage and GAPDH expression levels in MM1R cells treated with 1 µM withaferin a (WA) for 3 and 6 hrs. All data are presented as the mean ± s.d., n=3 biologically independent samples per treatment (*p < 0.05, **p < 0.01, ANOVA).

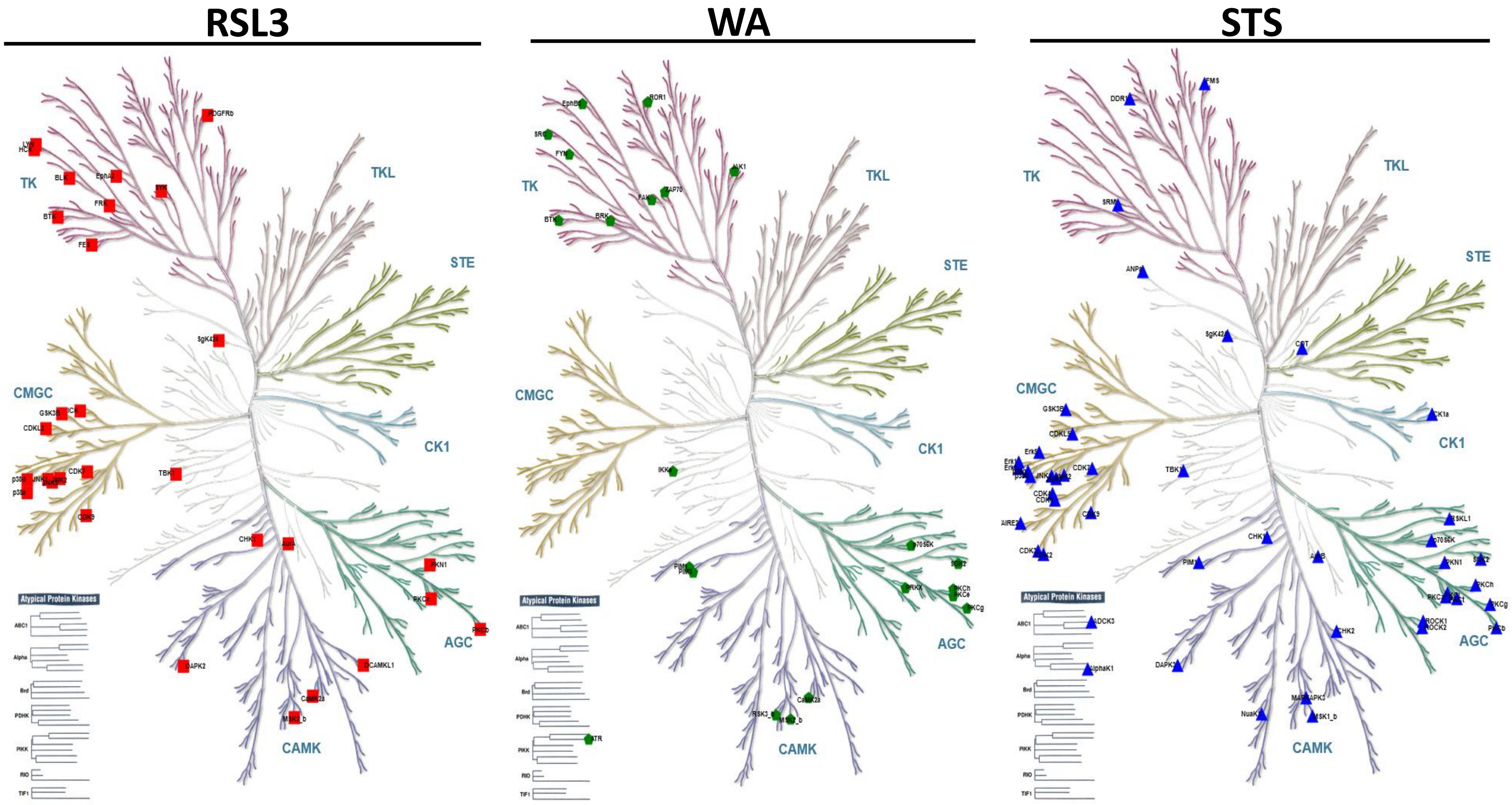

Figure S6: KinMap phylogenetic kinome tree. Kinases inhibited by RSL3 treatment are indicated by red squares (■) (left), kinases inhibited by STS treatment are marked as blue triangles (▲) (right), kinases inhibited by WA are indicated as green hexagons (⬢). Only kinases with a mean final score ≥ 1.3 are included in the graph. Abbreviations: TK, tyrosine kinase group ;TKL, tyrosine kinase-like group; STE, serine/threonine kinase group; CK1, casein kinase 1 group; AGC, protein kinase A, G, and C group; CAMK, Ca^{2+}/calmodulin-dependent kinase group; CMGC, cyclin-dependent kinase, mitogen-activated protein kinase, glycogen synthase kinase, and CDC-like kinase group.

Table S1. Original images western blot analysis

<table>
<tr><th>Figure</th><th>Blot(s) with molecular marker</th></tr>
<tr><td>1c: Western blot detection and quantification of PARP-1 cleavage and GAPDH protein expression levels after STS treatment in MM1R cells</td><td>α-PARP-1 (full length 116 kDa*, cleaved 81 kDa+) and α-GAPDH (37 kDa#)
marker: Benchmark Pre-stained protein standard (Thermo, #10748-010)
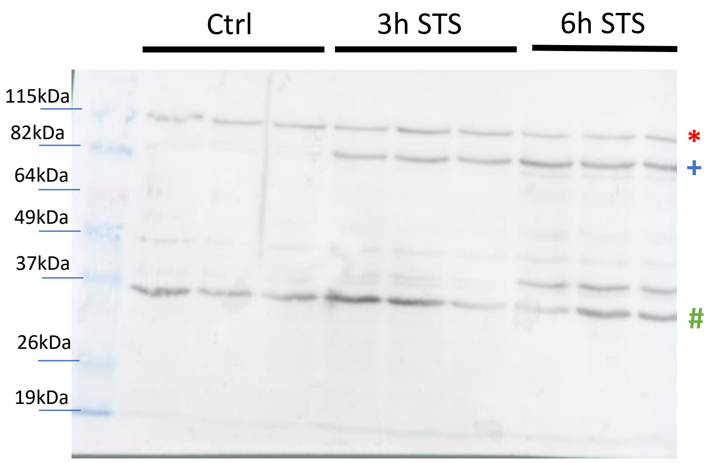
</td></tr>
<tr><td>S6: Western blot detection and quantification of PARP-1 cleavage and GAPDH protein expression levels after WA treatment in MM1R cells</td><td>α-PARP-1 (full length 116 kDa*, cleaved 81 kDa+) and α-GAPDH (37 kDa#)
marker: Precision Plus Protein All Blue prestained protein standard (Bio-Rad, #1610373)

α-PARP-1
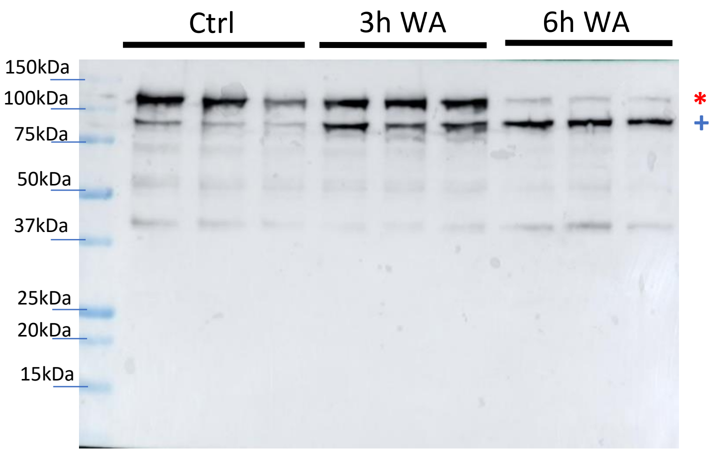
</td></tr>
</table>

	α-GAPDH<br>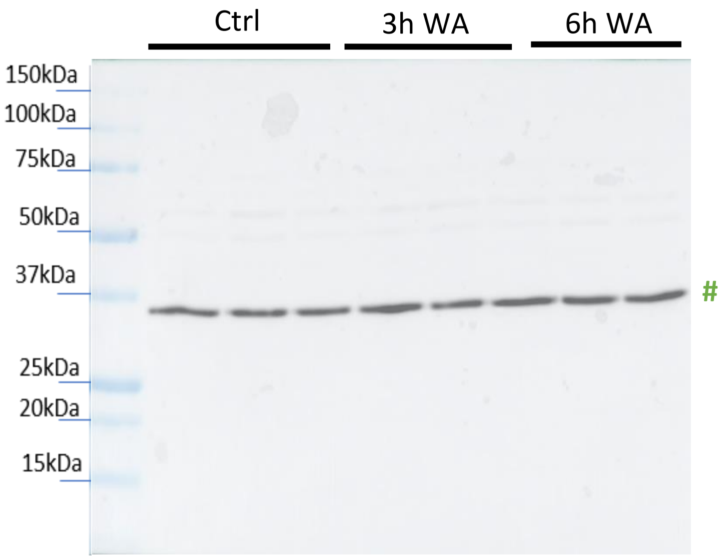
**S1:** Western blot detection and quantification of PARP-1 cleavage and GAPDH protein expression levels after RSL3 treatment in MM1R cells	α-PARP-1 (full length 116 kDa*, cleaved 81 kDa+) and α-GAPDH (37 kDa#)<br>*marker:* Benchmark Pre-stained protein standard (Thermo, #10748-010)<br>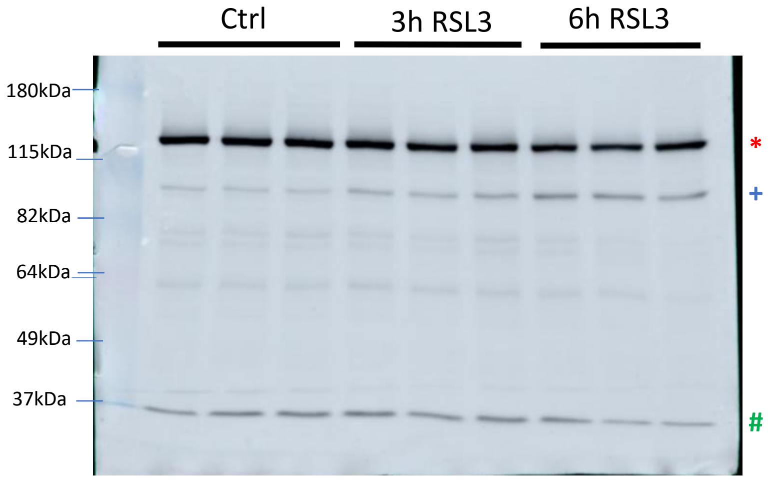
